# Crowdsourcing Attitudes and Beliefs About Upper Extremity Donation in the United States

**DOI:** 10.7759/cureus.59746

**Published:** 2024-05-06

**Authors:** Siam K Rezwan, Joseph S Puthumana, Gerald Brandacher, Carisa M Cooney

**Affiliations:** 1 Department of Plastic and Reconstructive Surgery, Johns Hopkins University School of Medicine, Baltimore, USA

**Keywords:** vascularized composite allotransplantation, public awareness, service members, veterans, hand donation, upper extremity donation, hand transplantation, upper extremity transplantation, vca, donation attitudes

## Abstract

Introduction

To date, upper extremity transplantation (UET) is the most frequently performed vascularized composite allotransplantation (VCA). Perceptions regarding upper extremity donation among Americans, particularly in veterans and service members (VSMs), are largely unknown.

Materials and methods

We administered a one-time survey to United States (US)-resident Amazon Mechanical Turk (MTurk) workers aged ≥18 years. Descriptive statistics were used to summarize study data; frequencies and percentages were calculated for categorical variables analyzed by Fischer’s exact test and using a two-tailed test assessing the statistical significance of p<0.05.

Results

A total of 860 respondents completed the study survey. Among these, 529 (61.5%) reported willingness to donate an upper extremity, 152 (17.7%) were undecided, and 179 (20.8%) were unwilling. A significantly higher proportion of those willing to donate were female (66.7%, p=0.009), non-Hispanic (63.9%, p=0.000), White (64.0%, p=0.004), non-religious (71.3%, p=0.001), not a VSM (62.8%, p=0.000), or non-amputees (62.9%, p=0.000).

Conclusions

Our survey found that being female, non-Hispanic, White, non-religious, non-VSM, or non-amputee was significantly associated with donation willingness. These findings may help guide VCA programs, organ procurement organizations, and researchers in efforts to develop targeted educational materials to broaden the public’s knowledge and awareness of VCA donation to further benefit all patients in need of or desiring transplantation.

## Introduction

Vascularized composite allotransplantation (VCA) is a groundbreaking, quality-of-life enhancing intervention involving the transplantation of multiple tissue types, such as the skin, blood vessels, muscles, bones, and nerves as a single unit [1]. VCA may best be considered when conventional reconstruction is unable to restore form and function lost due to severe injury or disfigurement or which was never present due to congenital deformities [2]. Although VCA can be performed on multiple parts of the body (e.g., face, abdominal wall), upper extremity transplantation (UET) is the most frequently performed procedure to date [3]. This may be due to the prevalence of upper extremity amputation in the US population, which is currently estimated to be over 41,000 and projected to exceed 300,000 by 2050 [4]. UET may also have particular relevance for US veterans and service members (VSMs) due to the United States Armed Forces’ involvement in armed conflicts over the past 20 years, which put service members at higher risk of trauma and amputations [5,6]. For individuals who have lost one or both hands or arms below the shoulder, UET can restore a greater degree of form, function, and sensation that more closely matches the native extremity than conventional reconstruction [7].

Most VCA procedures such as UET are only possible through graft donation from deceased donors [8]. As of July 3, 2014, vascularized composite allografts have been regulated in the United States (US) under the US Department of Health and Human Services (DHHS) Organ Procurement and Transplantation Network (OPTN) Final Rule (42 CFR part 121) [9]. This change caused organ procurement organizations (OPOs) to formally require specific and explicit authorization consenting to VCA donation in order to preserve public trust regarding organ donation. The inclusion of this specific authorization requirement means that indicating that one is an organ donor on one’s state driver’s license/ID is insufficient to allow vascularized composite allograft procurement: specific authorization must be obtained from the donor or a surrogate decision maker. Given that UET depends on deceased donors, understanding public perceptions of VCA donation is critical to the field. However, limited literature exists assessing the general US population’s perceptions of upper extremity donation [10-12]. Similarly, few studies assessing VSMs’ and VSM affiliates’ perceptions of upper extremity donation have been published [10,13]. As VSMs are at higher risk of trauma and amputation due to armed conflicts, and conventional reconstructive surgeries often fall short of delivering acceptable outcomes, the US Department of Defense (DoD) has made significant contributions to advance the field of VCA transplantation via funding, research, and education with the goal of benefitting wounded VSMs [14]. VSMs may also have more favorable perceptions towards upper extremity donation due to potentially increased exposure to individuals who have experienced severe traumatic injuries, including upper extremity amputation [10].

For these reasons, we conducted a cross-sectional survey study of Amazon Mechanical Turk (MTurk) workers to characterize attitudes and beliefs pertaining to upper extremity donation in US residents, with a particular interest in US VSMs. Based on prior organ and VCA donation literature, we hypothesize that younger, female, more educated, and VSM-affiliated respondents would report a greater willingness to donate upper extremities than individuals who are older, are male, have completed fewer years of schooling, and do not have a VSM affiliation [10-12].

## Materials and methods

Study participants were recruited through Amazon MTurk, an online crowdsourcing marketplace used for data validation and research to recruit survey respondents and yield high-quality data. The MTurk platform allows members of the general public who have registered on MTurk (aka, MTurk “workers”) to receive modest payment for completing human intelligence tasks (HITs), such as surveys. Creators of HITs post tasks to MTurk where workers can self-select which tasks they want to complete. Workers are offered a list of HITs, including brief descriptions of each task and the amount they will be compensated upon completion [15]. Studies of MTurk workers have shown that they represent the US population relatively well, though they are more likely to be younger than 50 years of age and have a college degree [16]. Therefore, while MTurk survey results may not be fully generalizable to the US population, the current literature has concluded that MTurk is a reliable mechanism through which to conduct survey studies and is comparable to conventional methods [17]. Additionally, MTurk has been used previously to recruit participants for studies evaluating organ and VCA donation attitudes that were published in high-impact journals [18,19].

Inclusion criteria were that respondents be US residents aged ≥18 years and registered MTurk workers. Prior to posting the survey to MTurk, we used MTurk’s requester settings to selectively target workers who self-reported a VSM affiliation. Respondents received $0.15 compensation upon completion of the survey.

We developed a 28-item, study-specific survey based on similar studies previously published in the literature [10,11]. The survey was hosted on Qualtrics (Qualtrics, Provo, Utah) and included binary, Likert, and free-response questions. Questions pertained to sociodemographic factors, amputee status, military affiliation, knowledge of one’s religion’s stance on organ and tissue donation, awareness of UET, differences between solid organ and upper extremity donation, knowledge and opinions regarding one’s state driver’s license/ID organ donor designation, perceptions of UET’s impact on quality of life (QOL), attitudes towards donating or receiving an upper extremity transplant, and willingness and attitudes towards organ donation and receipt (see Appendix, Figure 2). After opting into the survey, participants were provided with the study objective before beginning: “This questionnaire is for finding out people's opinions and beliefs on organ donation including hand/arm donation. Please read the general information below and answer the following questions.” The provided general information included descriptions of VCA and hand/arm transplantation, for whom it is intended, and the advantages and risks of upper extremity transplantation (see Appendix, Table 7). An artist’s rendition of the UET procedure was also provided in the introduction of the survey (see Appendix, Figure 2). The survey was reviewed by two subject matter experts (GB and CMC) before it was piloted among university faculty and students to assess face validity and ensure that respondents understood the survey questions as they were intended. Without compensation, students and faculty were asked to complete the survey that was distributed through a university announcement email list; a total of 144 respondents completed the pilot survey. As MTurk respondents are more likely to be younger, more educated, and have higher levels of internet literacy than the general population, this sample population was deemed a reasonable pilot test group [16]. The survey pilot test demonstrated evidence of face validity, and we revised two questions based on pilot sample responses.

All statistical analyses were performed using Statistical Product and Service Solutions (SPSS, version 26; IBM Corp, Armonk, NY). Descriptive statistics were calculated as frequencies and percentages for categorical variables and for classified free-text responses. Likert scale responses were displayed graphically by categorization of responses of 1-5 as disagree, 6-7 as agree, and 8-10 as strongly agree [20].

To enable analysis of free-response questions, one author (SKR) inductively coded responses by developing a coding classification system based on common themes for each free-response question. Response classifications were reviewed by a second author (CMC). Disagreements regarding classifications were resolved via open discussion.

Fisher’s exact test was used to compare respondents’ willingness to donate an upper extremity across individual demographic variables, including sex, age, race, ethnicity, religion, education level, employment status, annual household income, military affiliation, relative military affiliation, familiarity with religion’s stance on organ/tissue donation, and amputee status. We also selected Fisher’s exact test to accommodate categories with low response rates. Statistical significance was assessed at a two-sided significance of p<0.05.

The study was acknowledged as exempt from review by the Johns Hopkins Medicine Institutional Review Board (JHM IRB # IRB00233091) as no identifiable information was collected from respondents through the distribution or completion of the survey. The Checklist for Reporting Results of Internet E-Surveys (CHERRIES) was used as a guide for writing this manuscript [21]. Due to this study being conducted using MTurk and not as a typical internet survey for which CHERRIES was developed, it was not an ideal match for this study, so some checklist items were inapplicable and not reported.

## Results

Between 22 May and 22 July 2020, 860 respondents completed the study questionnaire. The majority of respondents were female (n=460, 53.5%), aged 18-35 years (n=498, 57.9%), non-Hispanic (n=678, 78.8%), and White (n=623, 72.4%); identified as Christian (n=521, 60.6%); and were not familiar with their religion’s stance on organ/tissue donation (n=454, 52.8%). The majority indicated having an education level of bachelor’s degree or higher (n=521, 60.6%), being employed (n=704, 81.9%), and having an annual household income of $50,000 or more (n=460, 53.5%). Participant demographics are summarized in Table 1.

**Table 1:** Sociodemographic table of respondent willingness to donate upper extremity (UE).

Variables	No, N=179 (20.8%)	Unsure, N=152 (17.7%)	Yes, N=529 (61.5%)	Total, N=860 (%)	p-value
Sex					
Male	91 (23.0)	86 (21.7)	219 (55.3)	396 (46.0)	0.009
Female	87 (18.9)	66 (14.3)	307 (66.7)	460 (53.5)	
Non-binary	1 (25.0)	0 (0.0)	3 (75.0)	4 (0.5)	
Age					
18-25 years	41 (24.4)	31 (18.5)	96 (57.1)	168 (19.5)	0.622
26-35 years	69 (20.9)	60 (18.2)	201 (60.9)	330 (38.4)	
36-45 years	36 (20.3)	32 (18.1)	109 (61.6)	177 (20.6)	
46-55 years	19 (16.1)	19 (16.1)	80 (67.8)	118 (13.7)	
56-65 years	7 (16.3)	4 (9.3)	32 (74.4)	43 (5.0)	
66-75 years	6 (28.6)	5 (23.8)	10 (47.6)	21 (2.4)	
76 years or greater	1 (33.3)	1 (33.3)	1 (33.3)	3 (0.3)	
Hispanic or Latino					
Yes	31 (17.0)	55 (30.2)	96 (52.7)	182 (21.2)	<0.001
No	148 (21.8)	97 (14.3)	433 (63.9)	678 (78.8)	
Ethnicity					
White	106 (17.0)	118 (18.9)	399 (64.0)	623 (72.4)	0.004
Black or African American	27 (30.3)	8 (9.0)	54 (60.7)	89 (10.3)	
American Indian or Alaska Native	6 (33.3)	3 (16.7)	9 (50.0)	18 (2.1)	
Asian	29 (29.3)	17 (17.2)	53 (53.5)	99 (11.5)	
Native Hawaiian or Pacific Islander	1 (50.0)	1 (50.0)	0 (0.0)	2 (0.2)	
Other	10 (34.5)	5 (17.2)	14 (48.3)	29 (3.4)	
Religion					
No religion	41 (17.3)	27 (11.4)	169 (71.3)	237 (27.6)	0.001
Christianity	110 (21.1)	108 (20.7)	303 (58.2)	521 (60.6)	
Islam	10 (43.5)	4 (17.4)	9 (39.1)	23 (2.7)	
Hinduism	1 (6.7)	2 (13.3)	12 (80.0)	15 (1.7)	
Judaism	5 (23.8)	4 (19.0)	12 (57.1)	21 (2.4)	
Buddhism	1 (9.1)	0 (0.0)	10 (90.9)	11 (1.3)	
Other	11 (34.4)	7 (21.9)	14 (43.8)	32 (3.7)	
Familiarity with Religion’s Stance on Organ/Tissue Donation					
Not familiar	103 (22.7)	71 (15.6)	280 (61.7)	454 (52.8)	0.144
Familiar	76 (18.7)	81 (20.0)	249 (61.3)	406 (47.2)	
Educational Level					
No formal education / some grade school	0 (0.0)	0 (0.0)	1 (100.0)	1 (0.1)	0.148
Some high school	2 (14.3)	4 (28.6)	8 (57.1)	14 (1.6)	
High school diploma	46 (24.9)	29 (15.7)	110 (59.5)	185 (21.5)	
Associate's degree	39 (28.1)	25 (18.0)	75 (54.0)	139 (16.2)	
Bachelor's degree or higher	92 (17.7)	94 (18.0)	335 (64.3)	521 (60.6)	
Employment Status					
Employed	137 (19.5)	131 (18.6)	436 (61.9)	704 (81.9)	0.066
Non-employed	42 (26.9)	21 (13.5)	93 (59.6)	156 (18.1)	
Annual Household Income					
Less than $50,000	96 (24.0)	78 (19.5)	226 (56.5)	400 (46.5)	0.034
$50,000-$99,999	54 (17.4)	59 (19.0)	198 (63.7)	311 (36.2)	
$100,000-$149,999	20 (19.8)	10 (9.9)	71 (70.3)	101 (11.7)	
More than $150,000	9 (18.8)	5 (10.4)	34 (70.8)	48 (5.6)	

Most respondents were non-VSMs (n=744, 86.5%), did not have any relatives who were VSMs (n=558, 64.9%), were not an amputee (n=770, 89.5%), and were not related to an amputee (n=696, 80.9%). Participant military affiliation and amputee status are summarized in Table 2.

**Table 2:** Willingness to donate upper extremity by military affiliation and amputee status.

Variables	No, N=179 (20.8%)	Unsure, N=152 (17.7%)	Yes, N=529 (61.5%)	Total, N=860	p-value
Military Affiliation (Veterans and Service Members)					
Yes	15 (12.9)	39 (33.6)	62 (53.4)	116 (13.5)	<0.001
No	164 (22.0)	113 (15.2)	467 (62.8)	744 (86.5)	
Relative Military Affiliation (Relative of Veterans and Service Members)					
Yes	52 (17.2)	66 (21.9)	184 (60.9)	302 (35.1)	0.023
No	127 (22.8)	86 (15.4)	345 (61.8)	558 (64.9)	
Living with Limb Loss					
Yes	13 (14.4)	32 (35.6)	45 (50.0)	90 (10.5)	<0.001
No	166 (21.6)	120 (15.6)	484 (62.9)	770 (89.5)	
Related to Someone Living with Limb Loss					
Yes	24 (14.6)	38 (23.2)	102 (62.2)	164 (19.1)	0.027
No	155 (22.3)	114 (16.4)	427 (61.4)	696 (80.9)	

**Figure 1:**
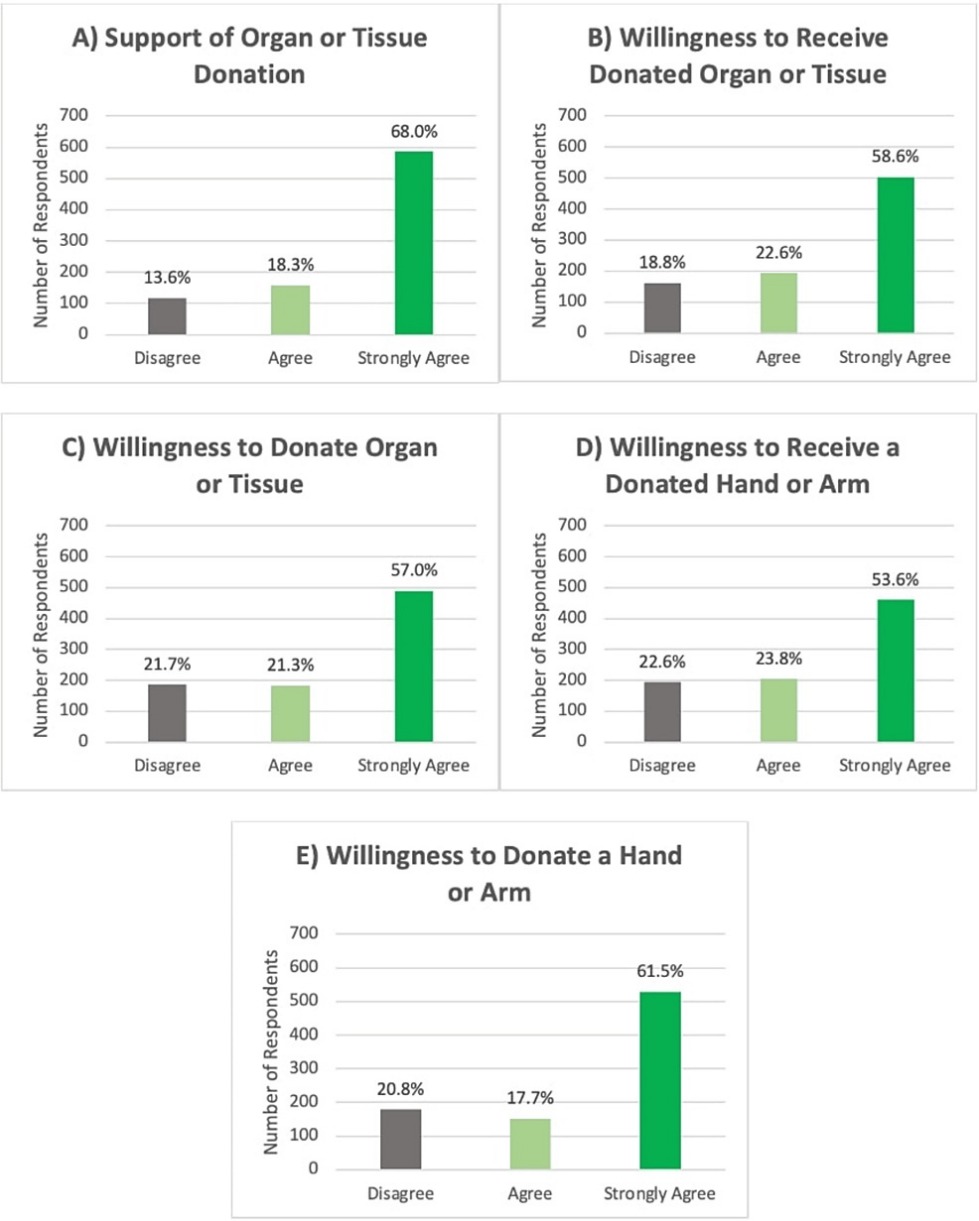
Respondents’ degree of agreement with statements (A-E) regarding organ/tissue and hand/arm donation and receipt. Answers provided on a 10-point Likert scale were categorized as 1-5=Disagree, 6-7=Agree, and 8-10=Strongly Agree.

**Figure 2:**
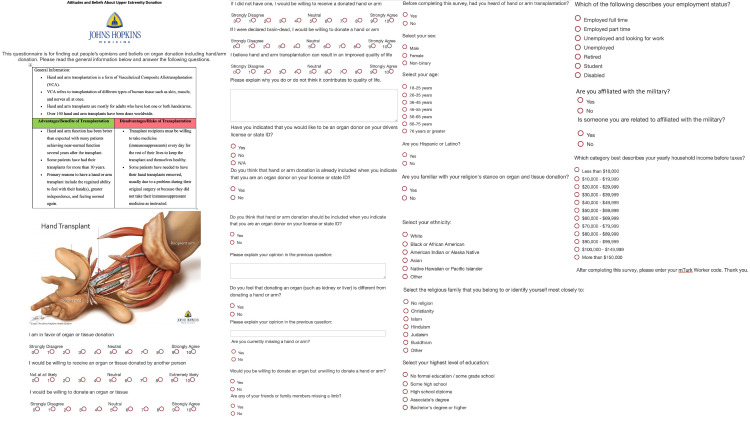
Study-specific survey hosted on Qualtrics and accessible via hyperlink on Amazon Mechanical Turk (MTurk). Image “Hand Transplant” embedded in survey copyrighted in 2012 by The Johns Hopkins Health System.

Of the total respondents, 179 (20.8%) were unwilling to donate an upper extremity, 152 (17.7%) were unsure, and 529 (61.5%) were willing to donate. Among those willing to donate, a significantly higher proportion were female (66.7%, p=0.009), non-Hispanic (63.9%, p<0.001), White (64.0%, p=0.004), non-religious (71.3%, p=0.001), non-VSMs (62.8%, p<0.001), and non-amputees (62.9%, p<0.001) (Tables 1-2). Most respondents supported organ/tissue donation (n=743, 86.3%) (Figure 1A), were willing to receive donated organs or tissues (n=698, 81.2%) (Figure 1B), and were willing to donate organs or tissues (n=673, 78.3%) (Figure 1C). Regarding hands and arms specifically, a majority of respondents were willing to receive (n=666, 77.4%) (Figure 1D) or donate (n=681, 79.2%) a hand or arm (Figure 1E).

When asked if they believed hand and arm transplantation can result in an improved QOL, most respondents either “strongly agreed” (n=585, 68.0%) or “agreed” (n=161, 18.7%), while 114 “disagreed” (13.3%). The most common explanations given by those who “strongly agreed” or “agreed” included perceptions of general improvement of QOL, improved functionality, greater feelings of normalcy, and more independence. The most common explanations for respondents who “disagreed,” while acknowledging a potentially improved QOL, addressed concerns associated with the challenges of UET, such as needing to take immunosuppressants, and discomfort with living with someone else’s hand/arm (Table 3).

**Table 3:** Attitudes and beliefs of upper extremity transplantation quality of life and reasoning. *Answers provided on a 10-point Likert scale were categorized as 1-5=Disagree, 6-7=Agree, and 8-10=Strongly Agree. **After entering their score, participants were asked to explain their opinions.

Provided prompt: I believe hand and arm transplantation can result in an improved quality of life	Disagree*, N=114 (13.3%)	Agree*, N=161 (18.7%)	Strongly Agree*, N=585 (68.0%)	Total, N=860
Themes Identified from Participant’s Free Responses** (Usable Responses, N=591) N (%)				
General improvement of quality of life/easier life	14 (6.2)	34 (15.1)	177 (78.7)	225 (38.1)
Improved functionality	6 (4.4)	17 (12.6)	112 (83.0)	135 (22.8)
Improved feeling of normalcy	0 (0.0)	10 (13.5)	64 (86.5)	74 (12.5)
More freedom and independence	0 (0.0)	3 (6.5)	43 (93.5)	46 (7.8)
Improvement of mental/emotional health	0 (0.0)	1 (4.8)	20 (95.2)	21 (3.6)
Difficulties with associated upper extremity transplantation challenges (e.g., immunosuppressants)	8 (44.4)	6 (33.3)	4 (22.2)	18 (3.0)
Other	9 (56.3)	3 (18.8)	4 (25.0)	16 (2.7)
Regained feeling	0 (0.0)	3 (21.4)	11 (78.6)	14 (2.4)
Concerns with facing the stigma of disabled people	1 (7.7)	2 (15.4)	10 (76.9)	13 (2.2)
Discomfort with having someone else’s upper extremity	6 (60.0)	2 (20.0)	2 (20.0)	10 (1.7)
General perceived decrease in quality of life/dislike of upper extremity transplantation overall	3 (37.5)	3 (37.5)	2 (25.0)	8 (1.4)
Prosthetics have less complications and equivalent or better functionality	2 (33.3)	3 (50.0)	1 (16.7)	6 (1.0)
Doubts of transplant efficacy (function and feeling restoration)	3 (60.0)	2 (40.0)	0 (0.0)	5 (0.8)
Excluded Responses, N=269, N (%)				
Unusable/nonsensical	24 (19.8)	44 (36.4)	53 (43.8)	121 (45.0)
Misunderstood question (e.g., “helps others”)	11 (12.5)	21 (23.9)	56 (63.6)	88 (32.7)
Unable to explain/unsure	25 (69.4)	3 (8.3)	8 (22.2)	36 (13.4)
Copy/pasted response	2 (8.3)	4 (16.7)	18 (75.0)	24 (8.9)

A total of 549 (63.8%) respondents indicated their willingness to be organ donors on their state IDs or driver’s licenses, and over half (n=449, 52.2%) believed that hand or arm donation is already included in this type of organ donation indicator. Among participants who are not organ donors per their state IDs, the majority believed upper extremity donation is not included (n=187, 70.0%) (Table 4).

**Table 4:** State ID organ donor indication and belief of upper extremity donation inclusion.

	Indicated as donor on ID, N=549 (%)	Did not indicate as donor on ID, N=267 (%)	N/A, N=44 (%)	Total, N=860 (%)
Believes upper extremity donation is included	320 (58.3)	80 (30.0)	11 (25.0)	411 (47.8)
Does not believe upper extremity donation is included	229 (41.7)	187 (70.0)	33 (75.0)	449 (52.2)

When asked if they thought hand or arm donation should be included when choosing to be designated as an organ donor, most participants responded “yes” (n=633, 73.6%). The most common explanations for their answers included believing/assuming that all body parts are included in the organ donor designation, general agreement that upper extremity donation should be included, and belief that upper extremity donation is the same as donating any other body part. Participants who thought upper extremity donation should not be included in one’s state ID donation consent (n=227, 26.4%) most commonly stated that it is “different” to donate a hand/arm compared to internal body parts, that upper extremity donation should require additional approval, and that hands/arms should not be considered organs (Table 5).

**Table 5:** Opinion of upper extremity donation inclusion and reasoning. *After selecting “Yes” or “No”, participants were asked to explain their opinions.

Do you think that hand or arm donation should be included when you indicate that you are an organ donor on your license or state ID?	Yes, N=633 (73.6%)	No, N=227 (26.4%)	Total, N=860
Themes Identified from Participant’s Free Responses* (Usable Responses, N=634), N (%)			
All body parts should be included in the donation/assumed all body parts included	144 (30.0)	18 (11.7)	162 (25.6)
It is “different” donating a hand/arm compared to internal body parts/upper extremity should require additional approval/“hands/arms are not organs”	58 (12.1)	72 (46.8)	130 (20.5)
Should be included – general	86 (17.9)	15 (9.7)	101 (15.9)
Donating an upper extremity is the same as donating any other organ	60 (12.5)	1 (0.6)	61 (9.6)
It would help others/“save life” of others/improve the quality of life of others	46 (9.5)	2 (1.3)	48 (7.6)
No longer used/needed after death and would help others	36 (7.5)	1 (0.6)	37 (5.8)
Should not be included – general	7 (1.5)	25 (16.2)	32 (5.0)
Other	18 (3.8)	11 (7.1)	29 (4.6)
Undecided – previously unaware of upper extremity donation, believes few others are aware of upper extremity donation, or that upper extremity donation is not typically considered	10 (2.1)	8 (5.2)	18 (2.8)
Do not want upper extremity to go to waste	15 (3.1)	0 (0.0)	15 (2.4)
General discomfort of using/donating another person’s upper extremity	0 (0.0)	1 (0.6)	1 (0.2)
Excluded Responses, N=226, N (%)			
Nonsensical response	84 (54.9)	37 (50.7)	121 (53.5)
Appeared to misunderstand the question	46 (30.0)	19 (26.0)	65 (28.8)
Unable to explain/unsure	13 (8.5)	16 (21.9)	29 (12.8)
Copy/pasted response	10 (6.5)	1 (1.4)	11 (4.9)

Nearly half (n=429, 49.9%) of respondents felt that donating an organ such as a kidney or liver was different from donating a hand or arm. The most common explanations for this among all respondents were that extremities are an external/visible part of the body (n=86, 13.3%), upper extremities are not required to live although their absence affects one’s QOL (n=64, 9.8%), and a general opinion that upper extremity donation is different from solid organ donation (n=61, 9.4%). For respondents who perceived hand/arm donations as the same as solid organ donation, the most common explanations were that these are all components of the body (n=232, 35.7%), they hoped the donation would benefit others (n=66, 10.2%), and there is no use for these body parts after death (n=51, 7.9%) (see Appendix, Table 6).

## Discussion

The fields of VCA and UET depend on the generosity of donors and their families; understanding perceptions of upper extremity donation is vital to the field’s development as UET grows as a viable treatment option for upper extremity amputees. In this cross-sectional survey study characterizing attitudes and beliefs regarding upper extremity donation in US residents with a particular interest in US VSMs, we found that, among those willing to donate their upper extremities (61.5% of respondents), a significantly higher proportion were female, non-Hispanic, White, non-religious, non-VSMs, who were not living with limb loss, and who were not related to someone living with limb loss.

Our findings are similar to the US Department of Health and Human Services’ 2019 National Survey of Organ Donation Attitudes and Practices [12]. This study, which surveyed 10,000 adults aged ≥18 years and included limited questions on hand donation, reported that 64.0% of respondents were willing to donate their hands after death [12]. Additionally, they found that non-Hispanic, White, and more educated respondents were more willing to donate their hands. While our study did not find respondent education level to be a significant factor in upper extremity donation willingness, we did find that women are more willing than men to donate their upper extremities. A Canadian survey by Lafreniere et al. similarly reported that female organ donor respondents were more willing to donate hands compared to male respondents [22]; these findings are confirmed by similar studies that found women to be more willing to donate their organs compared to men though the reasons for this remain unclear [23,24].

In surveying the role of religious beliefs in donation, we found that non-religious respondents were more willing to donate upper extremities than respondents who identified with a religion. Interestingly, being familiar with one’s religion’s stance on organ and tissue donation did not appear to impact the willingness to donate an upper extremity. The current literature remains unclear whether religion significantly impacts individuals’ willingness to donate organs or VCAs [25]. Since most religions do not prohibit organ donation or transplantation and promote the aid of others, education through religious organizations about organ and VCA donation may increase donation willingness among religious individuals [26].

Of the current literature that has assessed general VCA donation attitudes, one study focused exclusively on US military veterans [10]. While Ward et al. [10] demonstrated that most veterans are willing to donate VCAs such as upper extremities, our findings indicated that VSMs were less willing compared to non-VSMs. Notably, nearly one-third (33.6%) of VSMs were “unsure” compared to 15.2% of non-VSMs, suggesting a potential for targeting VCA donation education towards VSMs to increase donation willingness in the future. Additionally, we found no significant difference in upper extremity donation willingness based on whether respondents had relatives who were VSMs or not. Amputees were found to be slightly less willing to donate an upper extremity compared to non-amputees, but the lower willingness to donate an upper extremity by those living with limb loss may be explained by amputees not currently having an upper extremity to donate.

Similar to prior surveys on organ and VCA donation attitudes, most participants either supported or strongly supported organ and tissue donation [10-12,22]. Additionally, most participants “agreed” or “strongly agreed” that they were willing both to donate and receive organs or tissues including an upper extremity. Of note, more respondents were unwilling to receive a donated hand or arm (22.6%) than other donated organs or tissues (18.8%), suggesting a distinct difference in the perception of VCAs by a subgroup of the population.

The debate continues with respect to whether QOL improvements gained through upper extremity transplantation outweigh its risks and consequences such as the risk of rejection and the need for lifelong immunosuppressive therapy [27,28]. Overwhelmingly, this study’s participants agreed or strongly agreed (with 86.7% overall agreement) that UET can result in increased QOL. The most commonly provided explanations for this by respondents included general QOL improvement and increased normalcy, independence, and functionality. Only 2.4% of respondents mentioned regained sensation as a benefit of a transplanted upper extremity, a key difference between UET and currently available prosthetics [29]. Notable responses indicating reasons respondents were unwilling to receive a UET included expressions of discomfort at having someone else’s upper extremity, doubting the procedure’s efficacy, and a belief that prosthetics have fewer complications and equivalent or greater function.

Two of our findings were very similar to a study published in 2021 by the Health Resources and Services Administration (HRSA) in which they found that, despite 90% of adults supporting or strongly supporting organ donation in the US, only 60% are registered as organ donors [30]. Our study sample responded similarly with 86.3% of respondents supporting or strongly supporting organ or tissue donation, while only 63.8% were registered as organ donors on their state-issued ID. Our study’s results also highlight a considerable lack of knowledge regarding VCA donation authorization: despite VCA donation requiring specific, additional authorization from a donor’s surrogate decision-maker, nearly half of the respondents believed that hand or arm donation was already included when choosing to become an organ donor on one’s state-issued ID. When respondents were asked if upper extremity donation should be included in the state ID organ donor designation, 73.6% of respondents believed it should. This suggests that the public may be more accepting of broader authorization of VCA donations than originally anticipated by the Organ Procurement and Transplantation Network (OPTN)/United Network for Organ Sharing (UNOS) Vascularized Composite Allograft (VCA) Committee.

Approximately 25% of all respondents believed that all body parts either should be or assumed they be included when one elects to be an organ donor. One reason respondents thought that upper extremity donation should be included was not wanting their hand or arm to “go to waste” after they died. As might be expected, one reason many respondents thought that upper extremity donation should not be included was a perceived “difference” between “external” or “visible” body parts compared to solid organs. Interestingly, additional reasoning included that upper extremity donation may not typically be considered by the public and therefore should require additional approval. The “visibility” of upper extremities also connects to responses when participants were asked if they believed that donating an organ such as a kidney or liver is different from donating a hand or arm: respondents were equally divided (49.9% vs 50.1%). Over half of those who believed that there was no difference expressed that they are all parts of the body and are essentially the same. Those who believed there was a difference most commonly identified the key difference as upper extremities being external parts of the body that, unlike solid organs, are not required to live but instead affect QOL.

This study had several limitations. MTurk workers are compensated upon survey completion, so it is possible that some respondents aimed to complete the survey as quickly as possible to maximize their number of completed tasks and therefore profit, causing them to select answer choices without expressing their true thoughts or answering honestly. In response to free text questions, a small number of identical responses were observed, which indicates the possibility that some MTurk workers may have completed the survey twice; however, unique worker IDs are assigned to each MTurk user, which should prevent completion of the survey more than once. To help address this, our analysis included screening for and omitting random and nonsensical entries. While MTurk workers have been shown to reflect the adult (aged ≥18 years) US population relatively well, MTurk workers tend to be more educated than the general US population and are more likely to be <50 years of age [16]. Indeed, the percentage of respondents with a high level of education (bachelor’s degree or higher) in our study sample was 60.6%, almost double that of the US population. Despite these shortcomings, many studies have shown that MTurk workers do comprise a valid study population [18].

## Conclusions

Our Amazon MTurk survey found that being female, non-Hispanic, White, non-religious, non-VSM, and not being an amputee were factors significantly associated with willingness to donate an upper extremity. These data may be used to help identify groups less willing to donate their upper extremities in order to create educational materials or approaches to improve donation willingness. Examples may include images of and/or testimonials from transplant recipients on OPO materials and educational outreach performed through religious organizations or through veteran administration medical centers. Additionally, these data may provide specific insights regarding perceptions of upper extremity donation compared to solid organ donation; as many respondents thought that upper extremity donation was already included in opting in to being an organ donor on one’s state ID, additional, larger studies may be warranted to determine if VCA may be included in this opt-in in the future.

## Appendices

**Table 6:** Organ vs upper extremity donation opinion and reasoning. *After selecting “Yes” or “No”, participants were asked to explain their opinions.

Do you feel that donating an organ (such as kidney or liver) is different from donating a hand or arm?	Yes, N=429 (49.9%)	No, N=431 (50.1%)	Total, N=860
Themes Identified from Participant’s Free Responses* (Usable Responses, N=649), N (%)			
It is the same, all part of the body – general	22 (7.3)	210 (60.2)	232 (35.7)
Extremities (external/visible) are different than internal organs/tissues (internal/invisible)	78 (26.0)	8 (2.3)	86 (13.3)
Regardless will improve someone else’s life/will not be wasted	15 (5.0)	51 (14.6)	66 (10.2)
Upper extremity is not required to live, it affects quality of life	55 (18.3)	9 (2.6)	64 (9.8)
It is different – general	53 (17.6)	8 (2.3)	61 (9.4)
No use or need of upper extremity after death	3 (1.0)	48 (13.8)	51 (7.9)
Other	24 (8.0)	6 (1.7)	30 (4.6)
Upper extremity is not an organ	17 (5.7)	5 (1.4)	22 (3.4)
Alters appearance of dead body	16 (5.3)	1 (0.3)	17 (2.6)
More “personal” and part of identity	9 (3.0)	3 (0.9)	12 (1.8)
Upper extremity donation has different level of complexity than typical solid organ (e.g. kidney)	8 (2.7)	0 (0.0)	8 (1.2)
Excluded Responses, N=211, N (%)			
Nonsensical response	80 (62.0)	53 (64.6)	133 (63.0)
Appeared to misunderstand question	26 (20.2)	17 (20.7)	43 (20.4)
Unable to explain/unsure	7 (5.4)	12 (14.6)	19 (9.0)
Copy/pasted response	16 (12.4)	0 (0.0)	16 (7.6)

**Table 7:** Description of the upper extremity transplantation procedure provided to respondents.

General Information: Hand and arm transplantation is a form of Vascularized Composite Allotransplantation (VCA). VCA refers to the transplantation of different types of human tissue such as skin, muscle, and nerves all at once. Hand and arm transplants are mostly for adults who have lost one or both hands/arms. Over 140 hand and arm transplants have been done worldwide.	
Advantages/Benefits of Transplantation	Disadvantages/Risks of Transplantation
Hand and arm function has been better than expected with many patients achieving near-normal function several years after the transplant. Some patients have had their transplants for more than 10 years. Primary reasons to have a hand or arm transplant include the regained ability to feel with their hand(s), greater independence, and feeling normal again.	Transplant recipients must be willing to take medicine (immunosuppressants) every day for the rest of their lives to keep the transplant and themselves healthy. Some patients have needed to have their hand transplants removed, usually due to a problem during their original surgery or because they did not take their immunosuppressant medicine as instructed.
